# UVA-UVB Photoprotective Activity of Topical Formulations Containing *Morinda citrifolia* Extract

**DOI:** 10.1155/2014/587819

**Published:** 2014-07-15

**Authors:** Mairim Russo Serafini, Cassia Britto Detoni, Paula dos Passos Menezes, Rose Nely Pereira Filho, Vanessa Silveira Fortes, Maria José Fonseca Vieira, Sílvia Stanisçuaski Guterres, Ricardo Luiz Cavalcanti de Albuquerque Junior, Adriano Antunes de Souza Araújo

**Affiliations:** ^1^Departamento de Fisiologia, Universidade Federal de Sergipe (UFS), São Cristóvão, SE, Brazil; ^2^Programa de Pós-Graduação em Ciências Farmacêuticas, Universidade Federal do Rio Grande do Sul (UFRGS), Porto Alegre, RS, Brazil; ^3^Instituto de Tecnologia e Pesquisa (ITP), Aracaju, SE, Brazil; ^4^Faculdade de Ciências Farmacêuticas de Ribeirão Preto (FCFRP), Ribeirão Preto, SP, Brazil

## Abstract

Exposure to solar radiation, particularly its ultraviolet (UV) component, has a variety of harmful effects on human health. Some of these effects include sunburn cell formations, basal and squamous cell cancers, melanoma, cataracts, photoaging of the skin, and immune suppression. The beneficial photoprotective effects of topical formulations with the extract, *Morinda citrifolia*, have not been investigated. This present study aims to investigate the potential benefits of *M. citrifolia* topical application on the dorsal skin of mice, exposed to UVA-UVB light. Using 7 days of treatment, [before (baseline values) and 20 h after UV exposure], the thickness, skin barrier damage (TEWL), erythema, and histological alterations were evaluated. The results showed that the formulations containing the extract protected the skin against UV-induced damage.

## 1. Introduction

Ultraviolet radiation (UVR) from the sun is divided into UVC (200–290 nm), UVB (290–320 nm), and UVA (320–400 nm) [1]. Skin is the body organ that is most exposed to UVR. Human skin is constantly exposed to potentially harmful compounds and radiation because it serves as a protective barrier between the environment and its internal organs, thus making it liable to aging [2].

Photoaging is a long-term process that results from chronic exposure of the skin to solar UV radiation. UV radiation has been shown to cause a variety of lesions in DNA including pyrimidine dimmers of cyclobutane type, photoadducts of the pyrimidine, pyrimidine type, and other nucleic acid base photoproducts such a single-strand break and DNA-protein cross-links. These photoproducts are implicated in cellular lethality, mutation, and other biological effects [3]. Photoaging is characterized by thickening, roughness, coarse wrinkles, mottled pigmentation, and histologic changes, including damage to collagen fibers, the excessive deposition of abnormal elastic fibers, and an increase in glycosaminoglycans [4–6]. This phenotype is caused by alterations in cellular function, as well as deterioration of the extracellular matrix of skin connective tissue (dermis). The dermis provides structural support for the skin, and chronic solar UV exposure leads to fragmentation and disorganization of the primary structural protein elastin and collagen [7].

Several studies have evaluated the protective effect of natural products against damage induced by UVR in cells, tissues, animals, and humans. Photochemoprevention is the use of synthetic or natural substances that prevent, delay, or reverse the damage caused by UVR [8, 9]. Within this concept, we can find a variety of polyphenolic compounds with antioxidant, anti-inflammatory, immunomodulatory, and antimutagenic properties. In general, photochemopreventive agents act on two levels: (a) prevention of the damage caused by UVR and (b) modulation of different cellular responses to UVR that can prevent, stop, or correct tumor promotion and progression [1, 9, 10].

Free radicals and reactive oxygen species can be generated not only in metabolic reactions but also by UV radiation, especially by UVA (320–400 nm) radiation involving sensitized reactions. There are many endogenous chromophores in human skin which in the presence of UVA radiation can generate reactive oxygen species in the connective tissue of skin [3]. A decrease in the reactive oxygen species (ROS) load by efficient sunscreens and/or other protective agents may represent a promising strategy in preventing or at least minimizing ROS-induced cutaneous pathological states [11–13]. It has been suggested that the beneficial effects of topical antioxidants might be a successful strategy for diminishing UV radiation-mediated oxidative damage of the skin [14]. Thus, botanical antioxidants have shown good potential as photoprotective agents [15].

The tropical plant* Morinda citrifolia*, commonly known as “Noni”, is widely distributed in areas of Micronesia, Hawaii, Australia, and Southeast Asia. The genus* Morinda*, belonging to the family Rubiaceae, is indigenous to tropical countries and is considered an important traditional folk medicine [16, 17].

The fruits, roots, barks, and leaves of* M. citrifolia* have been used as folk medicine for the treatment of various illnesses, including burns, cancer, cold sores, skin inflammation, and wounds, among other things [18, 19].

In previous study, we demonstrated that the aqueous extract from* M. citrifolia* leaves contains alkaloids, coumarins, flavonoids, tannins, saponins, steroids, and triterpenoids and showed relevant* in vitro* antioxidant activity against different radicals. A carbomer gel base, containing the ethanol extract and juice pressed from the leaves, was evaluated in a UVB-induced erythema, and the UVB dose required to induce erythema was almost 3.5 times greater than with untreated skin [20]. Based on this data, we decided to investigate the potential benefits of topical application of aqueous extract from Morinda citrifolia on skin exposed to UVA-UVB light.

## 2. Material and Methods

### 2.1. Preparation of* Morinda citrifolia* Extract

*M. citrifolia* leaves were collected in São Cristóvão, Sergipe, Brazil [10°18′20.7′′ (S); 36°39′′7.2′′ (W)]. Herbarium voucher specimens (registry number 13503) were prepared and deposited at the Department of Biology, Federal University of Sergipe. The extract was prepared by boiling dry leaves in distilled water (7.5%; w/v) for 15 minutes; the solvent evaporated under reduced pressure and lyophilized.

### 2.2. Preparation of* Morinda citrifolia* Formulations

The* Morinda citrifolia* extract (0%, 10%, or 15% w/w) was dissolved in water (2.5 mL) and in ethanol (0.5 mL). Then, the formulation was suspended in hydroxyethylcellulose (2%). Imidazolidinyl urea (20 *μ*L) was added at the end. The controlled formulations did not contain the* Morinda citrifolia* extract (0%). The formulations were stored at 4°C.

### 2.3. Measurement of Spreadability

The spreadability of the formulations was determined by compressing the sample under several glass plates of known weight [21]. Twenty plates were subsequently placed over the sample at 1 min intervals. The spreading areas reached by the sample were measured in millimeters on vertical and horizontal axes. The results were expressed in terms of the spreading area as a function of the applied mass according to the following equation (1):

(1) $$\begin{array}{c} S_{i}=d^{2}\times \frac{\pi }{4} \end{array}$$

in which *S*_*i*_ is the spreading area (mm^2^) resulting from the applied mass *i*(*g*) and *d* is the mean diameter (mm) reached by the sample. The spreading area was plotted against the plate weight to obtain the spreading profiles. The experiment was repeated three times and the mean time was taken for calculation.

### 2.4. *In Vitro *Effectiveness

The formulations were scanned under UV light (200 to 400 nm) using a UV-1 800 PC spectrophotometer. Samples were placed in a quartz cuvette (model 4-Q, Hellma, Germany), presenting a capacity of 3 mL and an optical path length of 1 cm. To subtract the scattering of the gel matrix, a blank formulation (0% extract) prepared exclusively with hydroxyethylcellulose and ethanol/water was used as the baseline [22].

### 2.5. *In Vivo* Effectiveness

Male CF1 mice, weighing 20–30 g (*n* = 10* per group*), were housed in a temperature-controlled room, with access to water and food* ad libitum* until use. They were housed within cages with a 12 h light and 12 h dark cycle. All experiments were conducted in accordance with National Institutes of Health guidelines for the welfare of experimental animals and with the approval of the Ethics Committee of the Faculty of Pharmaceutical Science, Porto Alegre, Rio Grande do Sul (number 21804).

The animals were divided into five groups (*n* = 10): group 1 = nonirradiated control-NIC (the group was not irradiated and did not receive any topical treatment), group 2 = irradiated control-IC (the group was irradiated but did not receive any topical treatment), group 3 = irradiated and treated with the formulation 10%, group 4 = irradiated and treated with the formulation 15%, and group 5 = irradiated and treated with the vehicle-IV (formulation 0%).

Animals were anesthetized before formulation administration using intraperitoneal ketamine (80 mg/kg) and xilasine (10 mg/kg).

### 2.6. Formulation Administration

CF1 mice were randomly designed to different groups with 10 mice in each group. After the removal of dorsal hair (by trichotomy followed by 2 min exposure of the surface to Veet cream), the mice were topically treated on the dorsal surface (2 × 2 cm) with 0.1 g of formulations daily. The formulations were administrated 30 min before the dorsal surface irradiation. The untreated control groups irradiated and nonirradiated were included in the experiments.

### 2.7. Irradiation

The UVA-UVB source of irradiation consisted of a Repti Glo 10.0 lamp (Exo Terra) emitting a continuous spectrum between 270 and 400 nm 33% UVA and 10% UVB. The lamp was mounted 20 cm above the support where the mice were placed. The UVA-UVB output was measured using a model UV-400 Research Radiometer (I cel) with a radiometer sensor for UVA and UVB. The majority of the resulting wavelengths were in the UVB (290–320 nm; above 90%) and UVA (less than 10%) range and the peak emission was recorded at 314 nm. The mice were irradiated for 7 days, 2 h/day, with a daily dose of 1.9 J/cm^2^.

### 2.8. Assessment of Biophysical Skin Properties

The thickness of irradiated skin was measured by 7 days of treatment by dial thickness gage (Mitutoyo).

Transepidermal water loss (TEWL) was measured using a Tewameter R TM 210 (Courage & Khazaka Electronic GmbH, Germany) before (baseline values) and 20 h after UV exposure with 7 days of treatment. Readings of TEWL were registered in g/m^2^ *·*h during 2 min after 30 s probe equilibration on the skin. The UV-induced skin barrier damage, assessed by TEWL measurements, is due to abnormalities in the structures related to corneocytes adhesion and to disruption of the epidermal permeability barrier function [23].

Erythema was measured photometrically using a Mexameter R MX16 (Couragem & Khazaka Eletronic GmbH, Cologne, Germany) before (baseline values) and 20 h after UV exposure over 7 days. Six readings of the erythema index were recorded at each area at both points in time. The degree of erythema, quantified as an erythema index, is related to the hemoglobin content.

### 2.9. Animal Sacrifice

After treatment over 7 days, the mice were sacrificed by cervical dislocation 2 h after the ultimate UVA-UVB exposure. The full thickness of the dorsal skins was removed and fixed in 10% formalin until histological measurements.

### 2.10. Histological Measurements

Skin biopsies were fixed in 10% formalin, embedded in paraffin, according to routine protocol of histological procedures. Five micrometer thin sections of the paraffin-embedded skin were obtained and stained by means of hematoxylin eosin (HE) and Sirius red (SR) histochemical methods. Morphological analysis of the histological sections was performed by light microscopy followed by a closed numerical protocol in such a manner as the pathologist was not aware of which group was being evaluated until the end of the experiment.

### 2.11. Measurement of Cytotoxicity Activity

The B16F10 (murine malignant melanoma ATCC CRL-6475) cells were obtained from the Rio de Janeiro Cell Bank, Brazil. They were routinely grown in 150 cm^2^ tissue culture flasks in DMEM, supplemented with 1% (v/v) of an antibiotic solution containing 5 mg of penicillin, 5 mg of streptomycin, and 10 mg of neomycin per mL and 7.5% or 10.0% (v/v) heat-inactivated Fetal Bovine Serum at 37°C under 5% CO_2_.

The sensitivity of the cells to the* Morinda citrifolia* extract was determined by a standard spectrophotometric 3-(4,5-dimethylthiazole-2-yl)-2,5-diphenyltetrazolium bromide (MTT) assay [24]. Cells were seeded at a density of 10^5^ cells/wells into 96-well plates and incubated for 24 h at 37°C in an atmosphere of 95% air and 5% CO_2_. Then, 20 *μ*L of extract at different concentrations in a phosphate buffer saline (PBS) was added to the culture plates for 24 h. After treatment, cells were rinsed once with PBS and a serum-free culture medium without phenol red and were replaced in all wells. Cells were then incubated for 4 h with MTT solution (5 mg/mL).

The yellow tetrazolium salt was metabolized by viable cells to form purple crystals of formazan. The crystals were solubilized overnight (12 h) in a mixture consisting of 20% sodium dodecyl sulfate (SDS) in HCl (0.01 M). The product was quantified spectrophotometrically by measuring the absorbency at 570 nm using a microplate reader (*μ*QuantTM, BioTek Instruments Inc., USA). The cellular viability was expressed as a percentage of viable cells compared to the control group.

The percentage of viable cells was calculated as follows:

(2) %Viability =Total  number  of  viable  cellsTotal  number  of  viable  and  nonviable  cells×100.

### 2.12. Statistical Analysis

Data were expressed as mean ± standard errorof the mean (SEM) or ± standard deviation (SD). The data obtained was evaluated by one-way analysis of variance (ANOVA) followed by Tukey's test. Data analyses were performed using the GraphPad Prism 5.0 software. In all cases, differences were considered significant if *P* < 0.05.

## 3. Results

### 3.1. Measurement of Spreadability

The spreadability is an essential characteristic of semisolid pharmaceutical dosage forms for dermal application. Thus, the spreadability factor was measured (Table 1). The formulation containing the extract at 10% presented the higher spreadability (1.144 mm^2^ *·*g^−1^) against the formulation containing the extract at 15% (0.916 mm^2^ *·*g^−1^).

### 3.2. *In Vitro *Effectiveness

**Figure 1:**
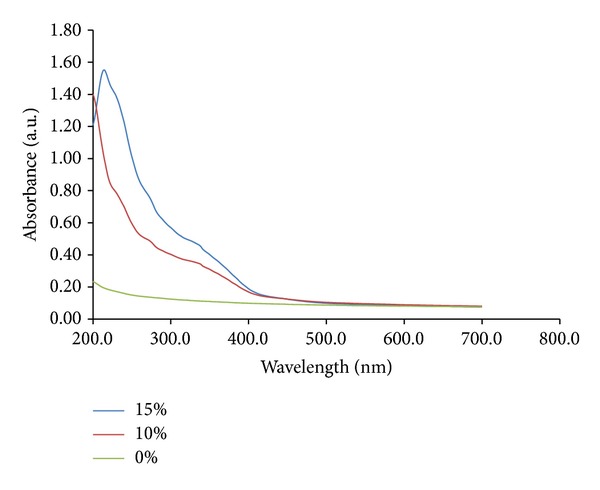
UV absorbance spectra of a blank formulation (0% extract), formulation extract 10%, and formulation extract 15%. The formulations were scanned under UV light (200 to 400 nm).

The absorbance spectrum of the formulations extracts (10% and 15%) (Figure 1) showed bands at 200 nm and at 240 nm and it showed absorbance in the UVA and UVB regions. The blank formulation (0% extract) did not show absorbance in this spectra.

### 3.3. Biophysical Skin Properties

**Figure 2:**
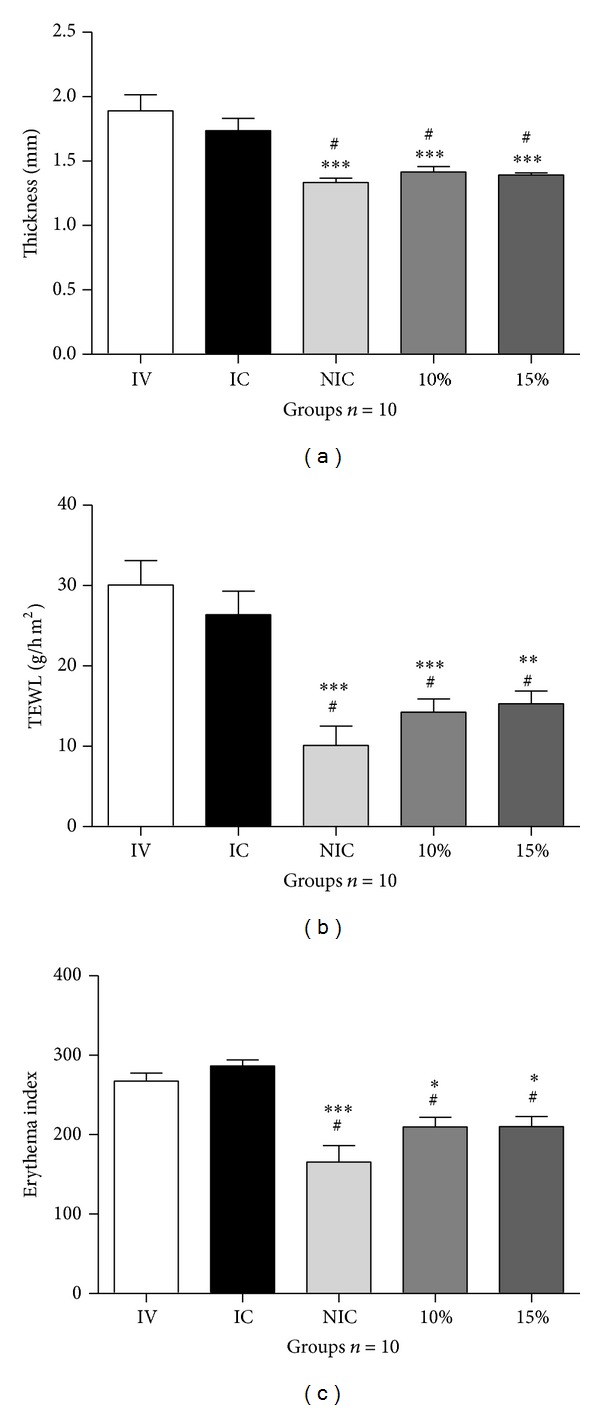
(a) Thickness values (mm), (b) transepidermal water loss, and (c) erythema obtained for the dorsal areas. IV = irradiated and treated with the vehicle (formulation 0%). IC = irradiated control (the group was irradiated but did not receive any topical treatment). NIC = nonirradiated control (the group was not irradiated and did not receive any topical treatment). 10% = group irradiated and treated with the formulation 10%. 15% = group irradiated and treated with the formulation 15%. The values were obtained before (baseline values) and 20 h after UV exposure during 7 days of treatment. (*n* = 10 mice). ^#^ *P* < 0.05 versus IC. ^∗^ *P* < 0.05, ^∗∗^ *P* < 0.01, and ^∗∗∗^ *P* < 0.001 versus IV. ANOVA, followed by Tukey's test. Data represents mean ± SEM.

The results showed that the thickness, TEWL values, and erythema indexes were significantly decreased by treatment with formulations containing the extract (Figures 2(a), 2(b), and 2(c), resp.).

### 3.4. Pathological Changes in the Skin Surface

**Figure 3:**
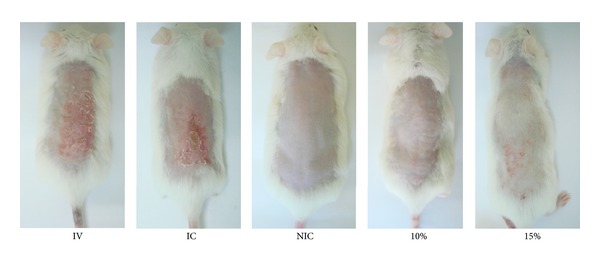
Skin biophysical techniques. Photoprotective effects of formulations on UVA-UVB-induced pathological changes in the skin surface on different groups after 7 days of treatment. IV = irradiated and treated with the vehicle (formulation 0%). IC = irradiated control (the group was irradiated but did not receive any topical treatment). NIC = nonirradiated control (the group was not irradiated and did not receive any topical treatment). 10% = group irradiated and treated with the formulation 10%. 15% = group irradiated and treated with the formulation 15%.

Treated areas with the formulation containing* Morinda citrifolia* extract (10 and 15%) have enhanced protection against UV radiation (Figure 3).

### 3.5. Histological Measurements

**Figure 4:**
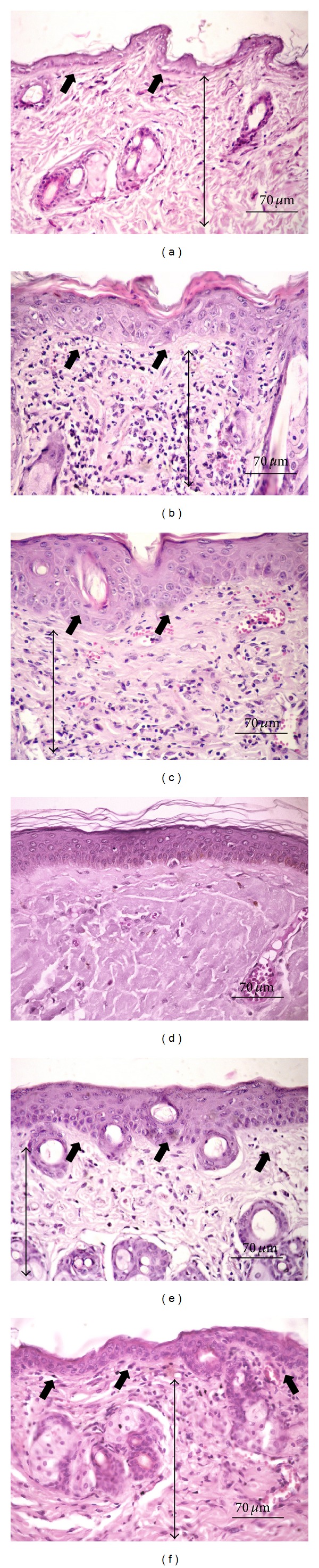
Hematoxylin-eosin stained histological sections. (a) Nonirradiated group-NIC showing thin epidermal lining and absence of dermal inflammation. (b) Irradiated-IC and (c) vehicle groups-IV presenting epidermal thickening and intense lymphocytic infiltration in the dermal tissue. (d) Elastotic change of the collagen. (e) 10% and (f) 15% formulations showing marked reduction of the inflammatory response. Note the thin epidermis in 15% formulation. Short thick arrows: epidermis. Long thin arrows: dermal connective tissues (Hematoxylin Eosin, 400x magnification).

*Hematoxylin Eosin (HE)*. As demonstrated in Figure 4(a), the nonirradiated group showed that the epidermis was represented by a thin, continuous, squamous, epithelial lining with an orthokeratotic surface. The connective tissue showed interlaced collagen fibers associated with spindle and round shaped stromal cells, consistent with fibroblasts. Some few capillary vessels and venules could also be seen, as well as numerous cutaneous appendages. No inflammatory infiltrates were found in the dermal tissues. Both irradiated and vehicle groups (Figures 4(b) and 4(c)) presented increased epidermal thickness associated to hyperorthokeratosis. The papillary dermis exhibited intense inflammatory infiltrate, mainly composed of lymphocytes, mild interstitial edema and prominent capillary, and venular hyperemia. Extensive areas of elastotic alterations of the collagen bundles (basophilic degeneration) were also observed throughout the connective tissue (Figure 4(d)). The treated groups presented remarkable reduction in the inflammatory content, as well as less expressive hyperemia. However, epidermal thickening was more evident in 10% than in 15% formulation (Figures 4(e) and 4(f)). Such similar histological features were quite similar to those verified in the normal dermal/epidermal tissues.

**Figure 5:**
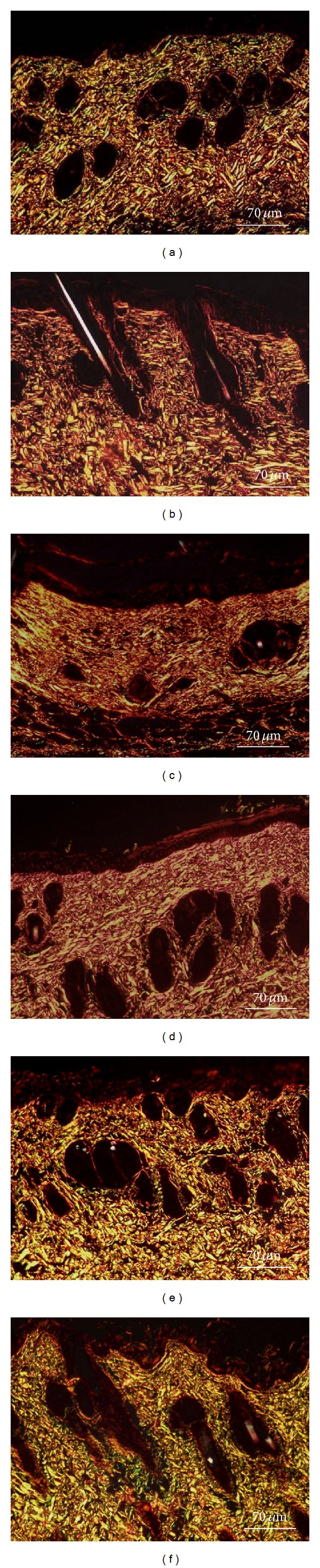
Sirius Red stained histological sections. (a, b) Nonirradiated-NIC groups presenting interlaced less compacted type I and III collagen fibers. (c) Irradiated-IC and (d) vehicle groups-IV showing dense disposition of parallel-arranged type I collagen fibers. (e) 10% and (f) 15% formulations presenting morphological and architectural collagen deposition pattern resembling that observed in the normal dermis (Sirius Red/Polarization Light, 400x magnification).

*Sirius Red (SR)*. The nonirradiated-NIC groups presented interlaced less compacted type I and III collagen fibers (Figures 5(a) and 5(b)). In irradiated and vehicle groups, the papillary dermis showed the presence of gross, thick, parallel-arranged collagen fibers, exhibiting intense yellow birefringence (type I collagen), which were associated to shorter and thinner reddish fibrils, perpendicularly disposed in relation to the surface. Such collagen bundles were densely compacted, showing sparse interfibrillar spaces (Figures 5(c) and 5(d)). Both treated groups (10 and 15% formulations) presented similar features regarding the collagenization pattern, showing a mixture of yellowish type I (predominant) and greenish type III collagen fibers. These fibers were more delicate, slightly wavy with variable thicknesses, lengths, and exhibited interlaced arrangements (Figures 5(e) and 5(f)). The interfibrillary spaces were more evident than in irradiated and vehicle groups. Such morphological appearances were very similar to those observed in nonirradiated group (Figures 5(a) and 5(b)).

### 3.6. Measurement of Cytotoxicity Activity

**Figure 6:**
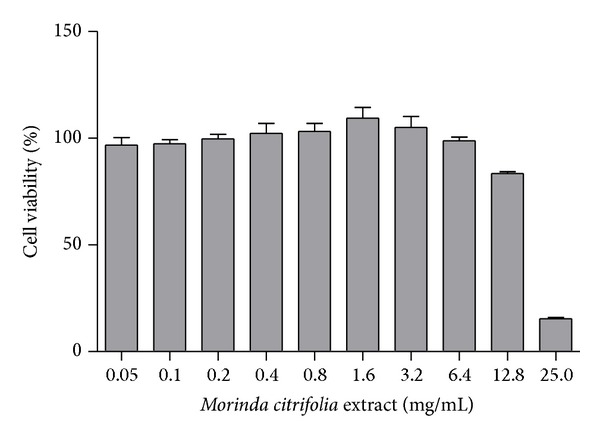
Cytotoxic effect of the* Morinda citrifolia* extract in B16F10 (murine malignant melanoma) cell line pretreated with the extracts at concentrations (0.05 to 25 mg/mL) for 24 h. Each value represents the mean ± SD.

The extract was evaluated on murine malignant melanoma (cell line B16F10) in order to examine their cytotoxic effects on malignant cells. Cytotoxicity of the extract on the growth of the B16F10 cell line is shown in Figure 6. Cell proliferation was analyzed 24 h after B16F10 cells had been cultured with the extract at different concentrations (0.05, 0.1, 0.2, 0.4, 0.8, 1.6, 3.2, 6.4, 12.8, and 25 mg/mL) using the MTT assay. The extract at concentrations of 0.05 to 12.8 mg/mL did not show any cytotoxic effects in the B16F10 cell line (showed no potential anticancer). A reduction in cell viability at high concentrations was observed (25 mg/mL).

## 4. Discussion

The most distinguished harmful effect of solar exposure is premature skin aging, especially UV irradiation. In recent years, thinning of the ozone layer has caused increased UV exposure. Detrimental effects of UV on the skin involve the generation of reactive oxygen species (ROS) [25]. Components of the skin, including DNA, lipids, and proteins, can be impaired by ROS, which is the cause of erythema, sunburn, immune suppression, and skin cancer [26]. Most of the disorders were formerly regarded as having been caused by UVB. Nevertheless, recent studies showed that UVA is also an essential factor [27].

Accordingly, use of antioxidants, capable of scavenging and quenching ROS, is another important approach to prevent UVB induced photoaging [27]. Other studies have shown that botanical compounds with an antioxidative activity are potential agents capable of reducing skin diseases including photoaging [28, 29].

The extract used in this work was the same studied by Serafini et al. [19] and showed relevant* in vitro* antioxidant activity against different radicals. The extract was chemically analyzed (qualitative method). The phytochemical screening of extract showed the presence of alkaloids, coumarins, flavonoids, tannins, saponins, steroids, and triterpenoids. Total phenolic content of the extract was 196.8 mg of phenolic equivalents (gallic acid) per gram of extract. Furthermore, qualitative analysis of extract was performed by HPLC, revealing the presence of 3 major compounds, all showing ultraviolet spectra typical of flavonols (quercetin-3-O-rutinoside or rutin and kaempferol glycosides). According to Deng et al., [30] flavonol glycosides, mainly rutin, are the major compounds in* M. citrifolia* leaves.

The presence of rutin in the formulation could also be secondarily involved in this process of collagenization. It has been demonstrated that the rutin is able to impair the glycation of collagen [31], a biochemical event involved in the pathogenesis of cell damaging in UV-irradiated tissue [32].

The use of botanical supplements to protect the human skin from the adverse biological effects of solar radiation has received great interest in recent years [33]. However, most tests have been conducted by oral ingestion or topical application of these ingredients in inappropriate vehicles, which can alter the skin barrier. To be of practical use to dermatologists and to be safe and efficient, extracts must have physicochemical characteristics that permit their incorporation in adequate concentrations in a skin-compatible vehicle (e.g., gels, moisturizing creams) [34]. In this study, the capacity of the* Morinda citrifolia* extract, added to topical formulations (gels), to prevent or to treat UVA-UVB irradiation-induced skin damage in mice was evaluated.

First, three formulations presenting different concentrations (0%, 10%, and 15%) were evaluated* in vitro* by spectrophotometer. UV absorbance spectra showed peaks at 200 nm and at 240 nm (UVC region) and absorbance in the UVA (320–400 nm) and UVB (290–320 nm) regions. The blank formulation (0% extract) did not show absorbance in this spectra. These data are compared to commercial organic filters. Thus, this result suggests a photoprotective effect of the extract. The concentration of formulation (10% and 15% w/w) used in this assay was selected based on a pilot study.

Second, the gel formulation efficacy against UVA-UVB induced skin damage was evaluated* in vivo *by measuring the following parameters: thickness values (mm), transepidermal water loss (TEWL), and erythema.

Moisturizing, antifree radicals, and sunscreen products have been used largely to prevent skin disorders resulting from UV damage. However, other aspects of skin damage should be considered, such as erythema, sunburn cell formation, epidermal hyperplasia, and impairment of the skin barrier integrity, which in turn is related to skin dryness, skin aging, and increased incidence of irritant contact dermatitis [35].

UV radiation damages the structures responsible for corneocyte adhesion and the functioning of the permeability barrier, which in turn alter thicknesses and TEWL measurements and accelerate the desquamation process [36].

The thickness, TEWL, and erythema were observed in our study and only the formulations containing* M. citrifolia* extract protected mouse skin against the UV-induced damages. The pronounced protective effects provided by both formulations (10 and 15%) in the three parameters measured were not due to any extraneous raw material but were shown by the unprotected groups treated by vehicle only (VI). Furthermore, the results show that areas treated with this extract (10 and 15%) were statistically equivalent to the unirradiated control ones (NIC), which represented the intact skin areas.

TEWL is an important parameter to be evaluated in studies of skin photoprotective effects, considering that UV irradiation of mammalian skin disrupts the epidermal permeability barrier function, accompanied by an increase in transepidermal water loss (TEWL) and alterations in the stratum corneum lipid profile [33].

In the TEWL measurement, the group 10% was different from IC, and the 15% was not. As well as in the thickness and TEWL, the protective effect of 10% was most pronounced. It can also be seen in the photos of the dorsal of the animal. This observationcan be explained by differences in the spreadability. Formulations containing extract at 10% presented the higher spreadability.

The spreadability is represented by the thickness of the film that the preparation leaves on the skin, an important feature in cosmetics [37]. Those producing thinner films, that is, higher spreadability, are naturally of greater interest [38].

Formulations containing extracts were shown to be effective also in the presence of erythema, another parameter often used to evaluate photoprotection. Extract formulations reduced erythema formation when compared with the irradiated control (IC). This statement corroborates [20], which demonstrated that the* M. citrifolia* leaf extracts mitigate the effects of UV light and provide some measure of defense against localized inflammation (topical microswelling).

Third, histological parameters were analyzed. Epidermal thickening and inflammatory changes are common histological changes observed in mice skin after short-term UV exposure [38, 39]. Such an increase in the epidermal thickness is supposed to occur in response to cell proliferation in attempt to regenerate the tissue, leading to a compensatory type of hyperplasia. However, the thickened epidermis as well as the thickened keratin layer (hyperkeratosis) could be observed in this study.

The presence of intense inflammatory infiltrate, elastosis and the accumulation of parallel-arranged type I collagen fibers observed in IC and vehicle groups have also been described as UV-induced dermal changes [31]. Inflammation is one of the major histological signs of tissue damage and has been reported in other studies as an early change of the connective tissue after UV exposure [38, 39]. On the other hand, elastosis represents an actinic-induced accumulation of amorphous elastin material replacing the normal dermis, unrelated to preexisting or newly synthesized collagen [40]. Moreover, it has been proposed that UV irradiation impairs ongoing collagen synthesis, primarily through the downregulation of types I and III procollagen expression [41]. Therefore, such impairment could likely promote delay in the collagen remodeling, leading to the typical immature parallel-arranged disposition of the fibers [31].

In this study, only 15% formulation avoided compensatory epidermal thickening. However, the reduction of the major deleterious effects associated with the UV exposure on the animals treated with both 10 and 15% formulations is strongly suggestive of photoprotective effect, regardless of the extract concentration.

Lastly, the extract was evaluated on murine malignant melanoma in order to examine its cytotoxic effects on malignant cells. A reduction in cell viability was observed only at the highest concentration (25 mg/mL). The results can suggest that the extract (in low concentrations) has only photoprotection effects and does not have antimelanoma activity.

## 5. Conclusion

In conclusion, present results strongly suggest the photoprotective effect of* M. citrifolia*. The results indicate that photoprotective effects are due to not only UV absorbance, like the UV filters, but also biological effects, which happened mainly with the application of the formulation containing the* Morinda citrifolia* extract. Further studies are necessary to clarify the precise nature of this photoprotective effect.

## Figures and Tables

**Table 1:** Measurement of spreadability of formulations containing *Morinda citrifolia* extract (10% or 15% w/w).

Formulations	∗SP1	∗SP2	∗SP3	∗SP Mean	∗SP Mean ± SD	C.V
Extract 10%	1.136	1.044	1.253	1.144	1.144 ± 1.07	9.4
Extract 15%	0.955	0.824	0.969	0.916	0.916 ± 0.08	8.7

*Spreadability factor (mm^2^ *·*g^−1^).
